# Retrieval of patients with severe respiratory failure on venovenous extracorporeal membrane oxygenation: an intensivist-led model

**DOI:** 10.1186/cc10702

**Published:** 2012-03-20

**Authors:** A Burrell, V Pellegrino, D Pilcher, S Bernard, M Kennedy

**Affiliations:** 1 The Alfred Hospital, Melbourne, Australia; 2 Adult Retrieval Victoria, Melbourne, Australia

## Introduction

Patients with severe respiratory failure may require venovenous extracorporeal membrane oxygenation (vv-ECMO). However, this treatment is only available in specialized centres. Previous reports of vv-ECMO cannula insertion and retrieval have included large teams of surgeons, perfusionists, physicians, retrieval doctors, paramedics and nurses. We hypothesized that an intensivist-led model for rapid response to a referring hospital, the insertion of vv-ECMO cannulae and subsequent retrieval would be safe and feasible.

## Methods

The Alfred Hospital ICU is the specialist centre for ECMO services for the states of Victoria and Tasmania in Australia. The intensivists in our ICU are trained to insert ECMO cannulae using a percutaneous femoral approach and manage the ECMO circuit during transport. A new ECMO retrieval service was set up in 2008 to allow the cannulation and retrieval of patients from other referring hospitals. The retrieval team comprises two intensivists to insert femoral cannulae and manage the ECMO circuit, a third physician to manage the ventilator and infusion pumps and a paramedic to manage the logistics of the patient transfer. We reviewed all consecutive patients from 2008 to 2011 with severe respiratory failure who received vv-ECMO and were retrieved to our specialist center.

## Results

There were 23 patients from 2008 to 2011. All cannulations were successfully performed percutaneously at the referring hospital by the intensivists. The underlying condition was H1N1 in 11 patients, bacterial pneumonia in six, acute lung injury in four, metastatic seminoma in one and multiple lung abscesses in one. The average age was 36 years (range 17 to 60 years). Males were 61%. Transport was by fixed-wing aircraft in 35% and road ambulance in 65%. The retrieval distance averaged 76 km (range 7 to 1,770 km). During transport, there were two transient pump failures requiring hand cranking and one monitor failure. These resulted in no adverse clinical effects. The average ICU length of stay was 14 days. Overall survival to hospital discharge was 17/23 (74%).

## Conclusion

An intensivist-led model of vv-ECMO cannulation and retrieval appears to be a safe and effective model for vv-ECMO retrieval. This model may lead to a more rapid and cost-effective response and is the subject of further study.

